# Regulatory Role of Sugars on the Settlement Inducing Activity of a Conspecific Cue in Pacific Oyster *Crassostrea gigas*

**DOI:** 10.3390/ijms22063273

**Published:** 2021-03-23

**Authors:** Mary Grace Sedanza, Hee-Jin Kim, Xerxes Seposo, Asami Yoshida, Kenichi Yamaguchi, Cyril Glenn Satuito

**Affiliations:** 1 Graduate School of Fisheries and Environmental Sciences, Nagasaki University, Nagasaki 852-8521, Japan; heejin@nagasaki-u.ac.jp (H.-J.K.); y-asami@nagasaki-u.ac.jp (A.Y.); kenichi@nagasaki-u.ac.jp (K.Y.); satuito@nagasaki-u.ac.jp (C.G.S.); 2 Institute of Aquaculture, College of Fisheries and Ocean Sciences, University of the Philippines Visayas, Miagao, Iloilo city 5023, Philippines; 3 School of Tropical Medicine and Global Health, Nagasaki University, Nagasaki 852-8523, Japan; seposo.xerxestesoro@nagasaki-u.ac.jp; 4 Organization for Marine Science and Technology, Nagasaki University, Nagasaki 852-8521, Japan

**Keywords:** *Crassostrea gigas*, Pacific oyster, conspecific cues, sugars, lectin, larval settlement

## Abstract

This study evaluated the larval settlement inducing effect of sugars and a conspecific cue from adult shell extract of *Crassostrea gigas*. To understand how the presence of different chemical cues regulate settlement behavior, oyster larvae were exposed to 12 types of sugars, shell extract-coated and non-coated surfaces, and under varied sugar exposure times. Lectin-glycan interaction effects on settlement and its localization on oyster larval tissues were investigated. The results showed that the conspecific cue elicited a positive concentration dependent settlement inducing trend. Sugars in the absence of a conspecific cue, *C. gigas* adult shell extract, did not promote settlement. Whereas, in the presence of the cue, showed varied effects, most of which were found inhibitory at different concentrations. Sugar treated larvae exposed for 2 h showed significant settlement inhibition in the presence of a conspecific cue. Neu5Ac, as well as GlcNAc sugars, showed a similar interaction trend with wheat germ agglutinin (WGA) lectin. WGA-FITC conjugate showed positive binding on the foot, velum, and mantle when exposed to GlcNAc sugars. This study suggests that a WGA lectin-like receptor and its endogenous ligand are both found in the larval chemoreceptors and the shell Ethylenediaminetetraacetic acid (EDTA) extract that may complementarily work together to allow the oyster larva greater selectivity during site selection.

## 1. Introduction

Larval settlement behavior and the choice of final substratum for attachment are important factors that influence survival, growth, and reproduction in benthic marine invertebrates [1,2,3]. The settlement behavior of larvae of the Pacific oyster *Crassostrea gigas* (Thunberg), like in other oyster species, often involves a swimming motion using its velum and with the foot extended forward, followed by a series of increasingly localized crawling maneuvers until it finds a suitable substratum [4,5,6]. During the search phase, the oyster larva makes use of its external chemoreceptors and transduces the cues it encounters in the environment into internal processes with neural and/or hormonal elements [3]. This is followed by a set of behavioral changes such as permanent cementation to the substratum where it finally metamorphoses into a juvenile [4]. Several studies have shown that chemical cues and surface properties of the substrate are important in settlement site selection by the larva [1,7].

The induction of oyster larval settlement by chemical cues in the marine environment may come from a variety of sources. A wide array of waterborne or surface-bound cues associated with conspecific adults have been reported to induce larval settlement. These include shells in *C. gigas* [8,9], *C. virginica* [10,11], *C. ariakensis* [12]; soft tissue homogenates in *Ostrea edulis* [13]; water pre-conditioned by adults of *C. ariakensis* [12], *C. gigas* [14], *C. virginica* [15,16,17], and *O. puelchana* [18]; oyster shell liquor or extrapallial fluid of *C. virginica* [19]; and ammonia [6]. Another source of such cues are chemicals bound to or released from bacterial biofilms in the form of extracellular polymeric substances [20,21,22,23]. The common components of extracellular and cell surface polysaccharides in bacterial biofilms are neutral sugars (D-glucose, D-galactose, D-mannose, D-fucose, L-rhamnose), amino sugars (*N*-acetylglucosamine and *N*-galactosamine), and some uronic acids [24,25,26]. However, despite the notable advances in this active field of study, much remains to be done in identifying the exact nature of these cues.

A previous report by Maki and Mitchell (1985) has demonstrated that lectins can be a useful tool in examining the role of sugar compounds in the larval settlement of marine organisms [27]. Lectins are sugar-binding proteins of non-immune origin that agglutinate cells and precipitate polysaccharides or glycoproteins [28]. Earlier works in other marine invertebrates, such as barnacles have shown that the presence of dissolved sugars influences the exploration, identification of conspecific cues, and larval metamorphosis [29,30,31]. Also, Vasquez et al. (2014) have provided evidence that a WGA-binding sugar chain in shells of conspecifics may mediate the settlement of *C. gigas* larvae on conspecifics, thus, highlighting the role played by lectin-glycan in biorecognition [8,9]. However, the nature of the chemical cue from shells of *C. gigas* is not yet fully characterized and elucidated. Understanding the processes and cues that lead to oyster larval settlement and metamorphosis can help us understand the underlying mechanisms that regulate population dynamics and chemical communication.

In this present study, the potential conspecific cue from adult shells of *C. gigas* was extracted using Ethylenediaminetetraacetic acid (EDTA) and its settlement inducing activity was evaluated. The suitable substrate for *C. gigas* shell EDTA extract (CgSE) for settlement assays was also determined. In nature, larvae also encounter different glycoproteins adsorbed to surfaces or exposed to dissolved cues. Hence, to understand how the presence of varied chemical cues regulate settlement behavior, larvae were exposed to 12 types of sugars including WGA-specific sugars (*N*-acetylglucosamine and *N*-acetylneuraminic acid) and exposed to conspecific crude shell extract (CgSE). We hypothesized that, during the search for suitable settlement sites, the carbohydrate moieties of chemical cues adsorbed in surfaces, as well as other waterborne cues come in contact with larval external chemoreceptors on the velum and foot. Therefore, the carbohydrate moieties could bind to lectins associated with these chemoreceptors and mediate the selection process. To test this hypothesis, we addressed the following questions: (1) Does the presence of dissolved low molecular weight sugar cues alone promote oyster larval settlement? (2) What types of dissolved sugars influence the ability of oyster larvae to discriminate suitable settlement sites in the presence of conspecific cues? (3) Does time exposure to sugar in the presence of a conspecific cue influence settlement? (4) Does blocking the carbohydrate moieties on the conspecific cue, i.e., adsorbed CgSE affect its settlement inducing activity? (5) Do lectins contained in the larval chemoreceptors of oysters bind dissolved sugars in the environment?

## 2. Results

### 2.1. Settlement Inducing Activity of Crassostrea gigas Shell EDTA (CgSE) Extract and Larval Substrate Specificity

**Figure 1:**
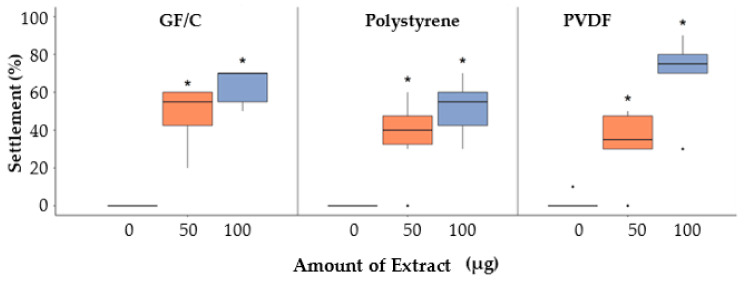
Settlement percentages of *C. gigas* larvae on different substrates coated with varying amounts of CgSE after 24 h. Asterisks (*) denote significant differences in amount coated on different substrates, using 0 µg as the baseline, determined via quasi-binomial glm (*p* < 0.05, *n* = 6, using different batches of larvae).

Settlement percentages of *C. gigas* larvae on different substrates coated with varying amounts of CgSE are shown in Figure 1. Among the variables, substrate type and amount of extract, the amount of extract was only found significant (Chi-square = 38.9; *p*-value < 0.001). The presence of adsorbed CgSE across all substrates showed a significant amount of extract dependent settlement inducing activity (*p* < 0.05). No settlement was observed on all substrates in the absence of CgSE. When substrates were coated 50 µg of CgSE, settlement ranged from 33 to 48%. Moreover, quasi-binomial glm analysis showed that the odds of settlement, if the amount is at 50 µg, is 57 times greater when compared to 0 µg CgSE (95% CI: 12.59 to 258.66; *p*-value < 0.001). At 100 µg, the mean settlement ranged from 52 to 70% in all CgSE-coated substrates. Hence, 50 µg CgSE was used as a suitable amount for the succeeding larval settlement assays. Shell chips at 50 mg were used as a reference substrate and settlement were at 35%.

### 2.2. Sugars and CgSE Effects on the Settlement of Oyster Larvae

**Figure 2:**
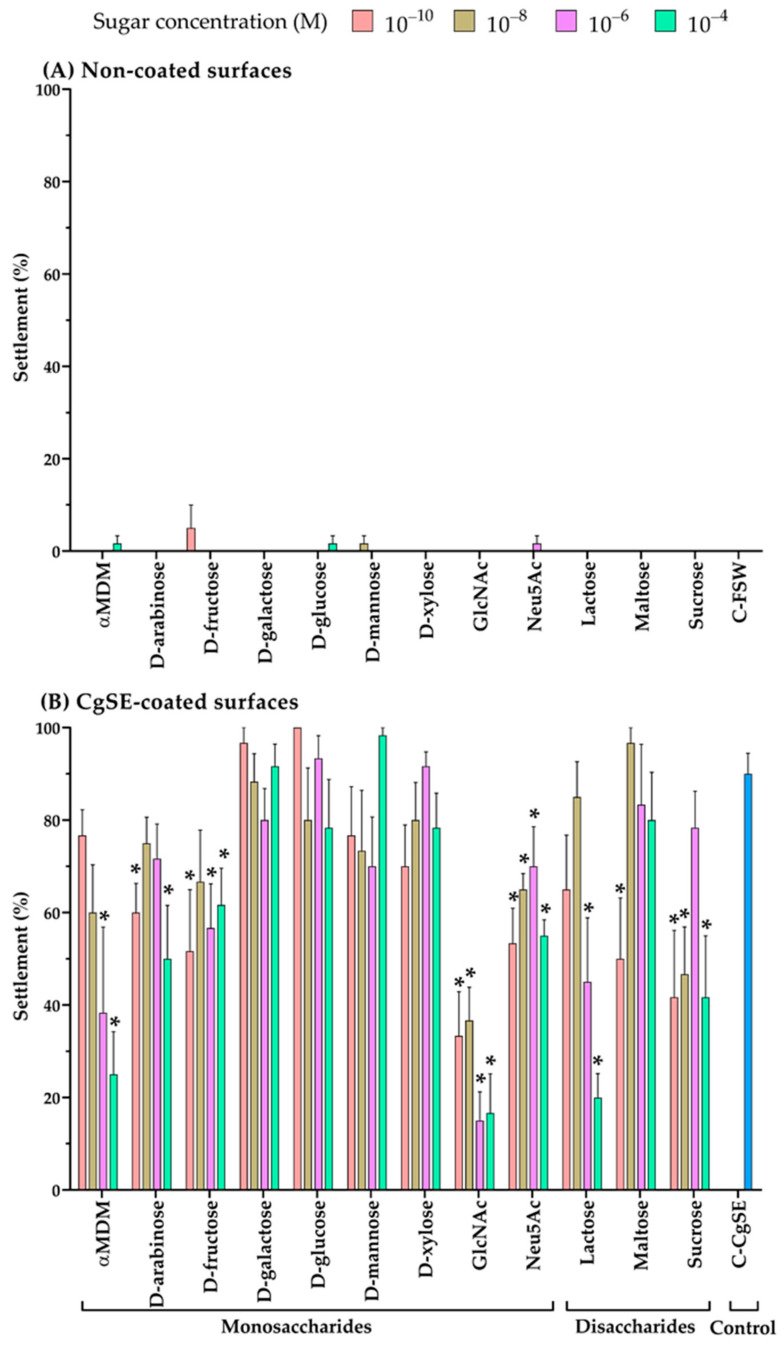
Settlement percentages of sugar treated *C. gigas* larvae exposed to different mono- and di-saccharides at different concentrations (10^−10^, 10^−8^, 10^−6^, 10^−4^ M). Settlement percentages in (**A**) non-coated surfaces and (**B**) CgSE-coated surfaces. Untreated oysters in filtered seawater (C-FSW) and untreated oysters exposed to shell extract only (C-CgSE) served as control. CgSE (50 µg) was applied to all coated surfaces. A sugar-specific statistical analysis was performed. Asterisks (*) indicate significantly different with respect to untreated oysters exposed to FSW alone (C-FSW, Figure 2A) and CgSE alone (C-CgSE, Figure 2B), determined via quasi-binomial glm (*p* < 0.05). Missing bars in the figure indicate no settlement. Data are the means (SEM) of six replicates using different batches of larvae.

Twelve sugars, which represent a wide range of possible carbohydrate moieties, associated with settlement-inducing chemical cues, reported in marine invertebrate larvae, were screened to test their effect on *C. gigas* larvae, and the results are shown in Figure 2. No settlement was observed in untreated *C. gigas* larvae on the multi-well with FSW (C-FSW). Settlement percentages of sugar treated, and untreated, larvae when exposed to non-coated surfaces and CgSE-coated surfaces elicited different responses (*p* < 0.05). In non-coated surfaces (Figure 2A), treatment of larvae with sugars alone did not elicit settlement except for some sugars (αMDM, D-fructose, D-glucose, D-mannose, and Neu5Ac). However, the settlement behavioral pattern was found to be similar when each of these sugars was compared to untreated larvae exposed to FSW alone (C-FSW, *p* > 0.05). No mortality was recorded in all sugar treated larvae. Larvae that did not settle after 24 h were actively swimming.

However, in the presence of a conspecific cue, CgSE-coated surfaces (Figure 2B), settlement responses were found similar to the control group (C-CgSE) when it was compared to larvae exposed to D-galactose, D-glucose, D-mannose, and D-xylose, irrespective of concentration differences (*p* > 0.05). On the contrary, an inhibitory effect on settlement was exhibited by GlcNAc and Neu5Ac-treated larvae across all concentrations when compared to the control group (C-CgSE, *p* < 0.05). GlcNAc-treated larvae showed the highest inhibition pattern among all the sugars. Moreover, larvae treated with other sugars showed both inhibiting and no effect depending on the concentration. Sugars and concentration levels that inhibited larval settlement were: αMDM at 10^−6^ and 10^−4^ M; D-arabinose at 10^−10^ and 10^−4^ M; D-fructose at all concentrations except at 10^−8^ M; lactose at 10^−6^ and 10^−4^ M; Maltose at 10^−10^ M; Sucrose at all concentrations except at 10^−6^ M (*p* < 0.05). No mortality was recorded in all sugar treated larvae. Sugar treated larvae that did not settle after 24 h exhibited closed-shell behavior.

### 2.3. Effect of Exposure Time to Sugars and CgSE on Oyster Larval Settlement Response

**Figure 3:**
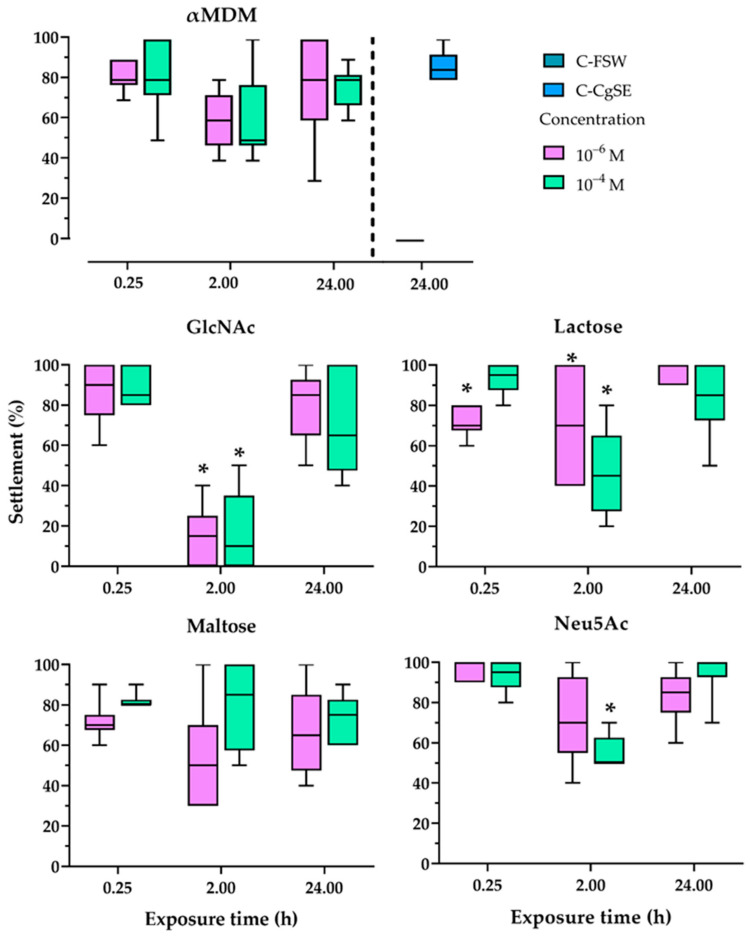
Settlement percentages of *C. gigas* larvae in response to different sugars and exposure times (0.25, 2, and 24 h) in the presence of CgSE. Following each exposure time, the larvae were thoroughly rinsed with filtered seawater and were incubated for 24 h in CgSE-coated wells. Larvae treated to a 24 h sugar exposure period was continuously immersed in CgSE-coated wells. Asterisks (*) indicate significantly inhibiting groups, determined via quasi-binomial glm (α= 0.05, *n* = 6, using different batches of larvae).

Settlement percentages of larvae exposed to sugars at short-duration (0.25 h), medium-duration (2 h), and prolonged-duration (24 h) in the presence of CgSE are shown in Figure 3. Based on the previous results on the sugar screening assay, some sugars that showed a significant inhibiting effect in the presence of a conspecific cue were selected to assess whether exposure time to a specific sugar influence settlement. A sugar-specific analysis was done to compare the effect on settlement when the larvae were exposed to different sugar concentrations and sugar exposure times in CgSE-coated surfaces. A high settlement percentage was recorded in untreated larvae exposed to CgSE alone (C-CgSE). All larvae that did not settle in this treatment were found actively swimming after 24 h. In sugar treated larvae, it is interesting to note that, while all showed similar low settlement patterns at 2 h exposure time, only GlcNAc-, lactose- and Neu5Ac-treated larvae were inhibited to settle. Sugar treatments on the larvae that were found inhibiting to settlement were GlcNAc at 2 h exposure at 10^−6^ and 10^−4^ M concentrations, lactose at 0.25 h exposure at 10^−6^ M as well as at 2 h exposure at both 10^−6^ and 10^−4^ M concentrations, and Neu5Ac at 2 h exposure at 10^−4^ M concentration (*p* < 0.05). In terms of behavioral observations, all sugar treated larvae irrespective of sugar type, exhibited closed-shell behavior upon initial contact with dissolved sugars and ceased any external movement. However, swimming activity eventually resumed after an hour of exposure. Moreover, all sugar treated larvae, irrespective of exposure time and concentration, that did not settle after the 24 h incubation period exhibited closed-shell behavior. No mortality was recorded in all treatments. No settlement was observed on untreated *C. gigas* larvae on the multi-well with FSW (C-FSW, blank control).

### 2.4. WGA Lectin Interaction with GlcNAc and Neu5Ac Binding: Effect on Oyster Larval Settlement

**Figure 4:**
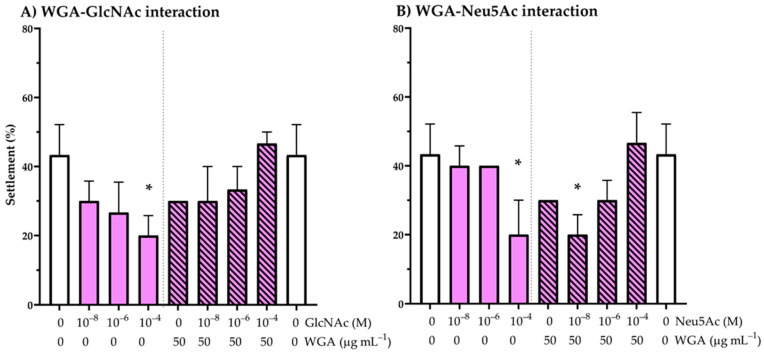
Settlement percentages in CgSE-treated GF/C filter papers when exposed to: WGA-binding sugars, (**A**) GlcNAc and (**B**) Neu5Ac, under varying concentrations of sugar treatment alone (shaded bars), and under WGA-GlcNAc or WGA-Neu5Ac mixture treatments (striped bars), for 2 h prior to assay. Adsorbed CgSE on GF/C filter papers alone served as a control (unshaded bars). Asterisks (*) denote a significant inhibiting effect on settlement, compared with other treatments determined via quasi-binomial glm (*p* < 0.05). Data are means (SEM) of three replicates.

The settlement percentages of larvae on CgSE treated- GF/C papers in the presence, or absence, of wheat germ agglutinin (WGA) lectin, and its binding sugars GlcNAc and Neu5Ac, are shown in Figure 4. Co-exposure of CgSE papers and different concentrations of GlcNAc or Neu5Ac alone (Figure 4A,B, shaded bars) showed an inhibiting effect on the oyster larval settlement at 10^−4^ M when compared to the control (CgSE paper only, unshaded bars). In treatments with the mixture of WGA, and where increasing concentrations of GlcNAc or Neu5AC were added to CgSE papers, both showed an increasing trend of settlement with concentration (Figure 4A,B, striped bars). Quasi-binomial glm analysis showed that settlement, in the presence of WGA alone or its mixture with GlcNAc in CgSE papers was similar for all treatments (*p* > 0.05), while in WGA-Neu5Ac interaction, an inhibiting effect on the settlement was found at CgSE-Neu5Ac-WGA (50 µg: 10^−8^ M: 50 µg) mixture treatment (*p* < 0.05).

### 2.5. WGA-Binding Distribution on C. gigas Oyster Larval Tissues

**Figure 5:**
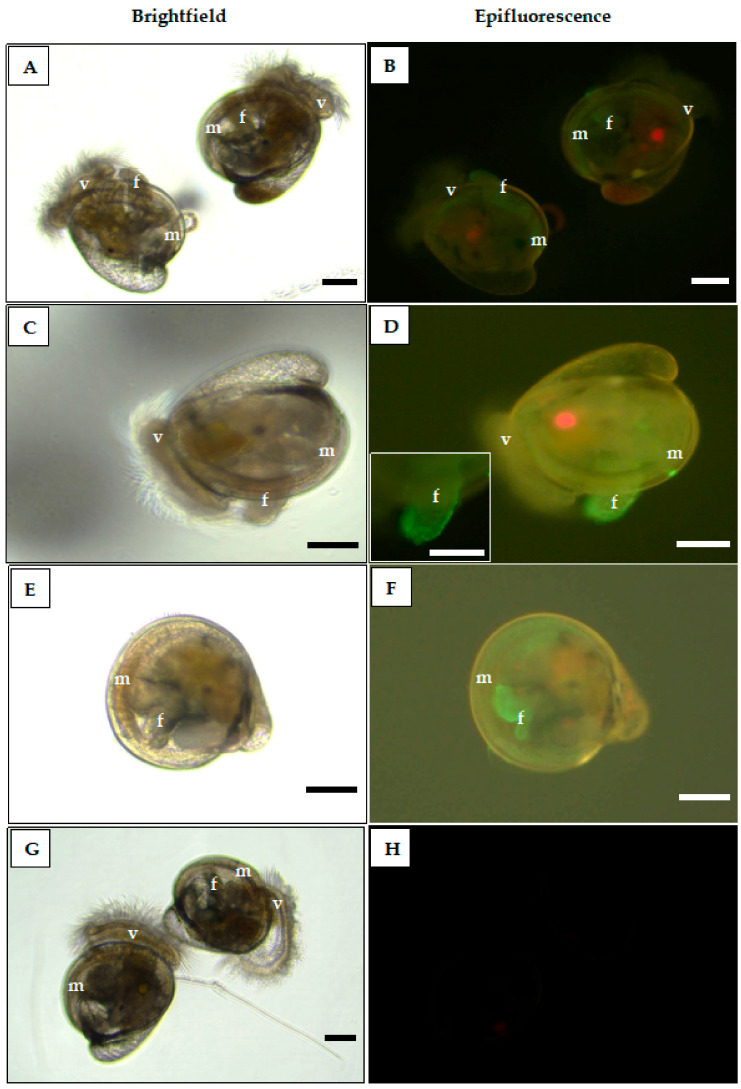
WGA binding to GlcNAc moieties on the mantle, foot, and velum tissues of *C. gigas* larvae stain green under epifluorescence view at 100× magnification and 200× magnification (plate **D** inset only). (**A**,**B**) GlcNAc sugar treated larvae at 10^−4^ M show weakly stained mantle, foot, and velum tissues. (**C**,**D**) GlcNAc sugar treated oyster larva at 10^−10^ M shows an intense binding stain on the mantle, velum, and foot tissues, as well as its cilia (inset photo). (**E**,**F**) Untreated oyster larva (positive control) with WGA-FITC conjugated lectin shows binding stain on the mantle and foot tissues. (**G**,**H**) Untreated larvae (negative control) without WGA-FITC staining indicates that the larvae do not exhibit autofluorescence. Abbreviations: f = foot, m = mantle, v = velum. Scale bar = 100 µm.

The results on the distribution of WGA-FITC binding sites on *C. gigas* oyster larval tissues are shown in Figure 5. Under epifluorescence view, GlcNAc-treated (10^−10^ and 10^−4^ M) larvae showed decreasing intensity of WGA-FITC conjugated lectin staining (green fluorescence) on the velum, mantle, and foot tissues as the sugar concentration increases (Figure 5B,D). Surfaces of cilia present on the foot also showed WGA lectin binding (Figure 5D). This supports the hypothesis that GlcNAc sugar may have bound to these tissues. However, untreated larvae exposed to WGA-FITC also showed positive staining on the mantle and foot tissues (Figure 5F). This may indicate that, even without GlcNAc sugar exposure, the evidence points to a WGA-binding sugar present on these tissues as well. No fluorescence was detected on the tissues of untreated larvae without WGA-FITC exposure, which proves the absence of autofluorescence in *C. gigas* larvae.

## 3. Discussion

Although the active search for suitable substrate or settlement sites, in response to a wide range of variables and cues, has been well-documented in oyster larvae [1,2,8,9,11,16,23,32], the exact mechanism(s) of how larval settlements are induced is not yet fully understood. Understanding these processes and cues can help us understand the underlying mechanisms that shape population structures and chemical signaling. In the present study, a conspecific cue from the *C. gigas* shell-EDTA extract showed a high concentration-dependent settlement, inducing activity comparable to that of the hydrochloric acid (HCl) extract, as reported by Vasquez et al. (2013) [8]. Furthermore, the selection of a suitable substrate, i.e., inert, and non-toxic, for analyzing settlement-inducing compounds is important. In this experiment, no larval settlement was elicited from all the tested substrates in the absence of CgSE. No mortality was also observed, indicating that these substrates indeed were inert and non-toxic to the larvae. The adsorption of CgSE on GF/C, Polystyrene, and PVDF substrates resulted in similar larval settlement responses, which indicate that any of these substrates are suitable for use. Relatively similar results were also reported by Vazquez et al. (2014)*,* where the highest number of post-larvae were recorded in GF/C and glass substrates [9]. Therefore, the direct application of the CgSE on polystyrene wells was used in the sugar screening and time exposure assays, while the GF/C filter papers were used in the lectin-sugar interaction assays. Fifty-microgram (50 µg) of CgSE was used for the succeeding larval settlement assays in this study.

Several studies have also shown that *C. gigas* larvae did not only settle through the mediation of conspecific cues but also settled in well-developed bacterial biofilms [20,23]. Bacterial biofilms are believed to possess chemicals bound to or released from in the form of extracellular polymeric substances [20,21,22,23]. The common components of extracellular and cell surface polysaccharides in bacterial biofilms are neutral sugars (D-glucose, D-galactose, D-mannose, D-fucose, L-rhamnose), amino sugars (*N*-acetylglucosamine and *N*-galactosamine), and some uronic acids [24,25,26]. The interactions between lectins and glycans have been implicated to play a critical role in biorecognition processes, such as in marine invertebrate larval settlement [9,33,34,35]. Herein, the results of this study demonstrate the influence of twelve types of dissolved sugars and a conspecific cue in *C. gigas* larval settlement. Larvae treated with different sugars in the absence of a conspecific cue, CgSE, did not settle. While, untreated larvae exposed to CgSE alone resulted in a high settlement response. The results in this study suggest that the presence of dissolved sugars alone could be a chemotactic attractant to the larvae, but it does not provide the necessary metamorphic cue. Earlier studies have shown that the larvae of *Crassostrea virginica* were attracted to bacterial metabolites, including glucose and amino acids, but were only induced to metamorphose in the presence of bacterial films [36]. Also, data in this present study indicates that induction of settlement in the larvae may not only require the presence of sugars alone, but also amino acids and other chemical groups, present at the site of chemical contact between the larvae and the substrate [37,38], as observed in the high settlement response on the control, CgSE alone (C-CgSE). This may also imply the need for multivalent interactions between multiple carbohydrate recognizing domains (CRDs) from lectins and multiple glycans that are often required to produce high-avidity binding interactions [38] for settlement induction to take effect. Hence, the high percentage of settlement on the conspecific cue, C-CgSE, could be in part, due to an increased avidity effect [38]. However, the precise mechanism of how lectins and glycans, in CgSE, enhance their avidity remains to be clarified. On the other hand, sugar treated larvae placed in CgSE-coated surfaces displayed varied settlement responses, most of which were found inhibitory (Figure 2B). One possible explanation for this could be that these low molecular weight sugars may have inhibited lectin-mediated processes as these sugars could have competed for the carbohydrate-binding sites on the CgSE or may have bound to the oyster larval lectin-like receptors [39]. In the present study, while several sugars were found inhibitory, their levels of inhibition were different, suggesting varying degrees of affinity of such sugars on the oyster larval chemoreceptors. Khandeparker et al. (2011) and Neal and Yule (1996), in separate experiments, inhibited cyprid larval settlement on surfaces with conspecific extract or the presence of barnacle species by exposing the larvae to different dissolved sugars. Their results indicated that sugars in solution can block the polar groups on the attachment disc through electrostatic adsorption of -OH groups [31,40]. Higher sugar concentrations block more polar groups, nullifying their contribution to adhesion, thereby, lowering adhesion thresholds below those for cohesive failure [40]. Moreover, compounds that inhibit the reaction examined at the lowest concentration is considered to have the highest affinity for the lectin and to be most complementary to its combining site [28]. Among those sugars in this study that were significantly inhibitory were α-methyl-D-mannoside (αMDM), D-arabinose, D-fructose, *N*-acetylglucosamine (GlcNAc), *N*-acetylneuraminic acid (Neu5Ac), and all disaccharides (lactose, maltose, sucrose). GlcNAc and Neu5Ac-treated larvae were significantly inhibited to settle on CgSE-coated surfaces at all concentrations suggesting that these sugars may have a very high affinity for the larval lectin-like receptor in the larvae. However, results in this study show that a lectin-like receptor in the larvae seems to bind with sugars from different specificity groups: hexose sugar (αMDM), pentose sugar (D-arabinose), ketose sugar (D-fructose), amino sugar (GlcNAc), sialic acid (Neu5Ac), reducing sugars (lactose, Maltose) and non-reducing sugar (Sucrose). Whether these sugars bind to the lectin on the same combining site or this lectin can combine simultaneously to different sugars, or exhibit ligand multivalency [28], remains to be clarified. On the other hand, D-galactose, D-glucose, D-mannose, and D-xylose-treated larvae were found to elicit similar responses compared to untreated larvae exposed to CgSE alone (C-CgSE). Also, in this study the influence of sugars—whether as monomers or dimers—and their concentrations have shown varying responses in oyster settlement. Many extracellular signal molecules act at very low concentrations (typically ≤ 10^–8^ M), and their receptors usually bind them with high affinity (dissociation constant Kd ≤ 10^–8^ M) [41]. The differential effects of sugar types on attachment to a substrate until settlement and metamorphosis suggests that the ratios of these sugars within a settlement inducing chemical cue could determine the maximum adhesion of a settling larvae on a substrata [40]. The effect of sugars on settlement are significant findings in the study of chemical communication and could have substantial implications for oyster ecology.

In this experiment, sugars that showed a concentration dependent and/or significant inhibiting effect in the presence of a conspecific cue were selected to assess whether exposure time to a specific sugar influence settlement. The results in this study demonstrate that the binding affinity of GlcNAc, Neu5Ac, and lactose sugars is influenced not only by concentration but also by exposure time. GlcNAc sugars showed the highest affinity, maximum inhibition occurring at 2 h exposure time at both 10^−6,^ and 10^−4^ M concentrations. Neu5Ac sugar show lesser affinity, maximum inhibition occurring at 2 h exposure time at 10^−4^ M concentration. While, lactose showed the maximum inhibitory effect at 0.25 h exposure at 10^−6^ M concentration, as well as at 2 h exposure, at both 10^−6^ and 10^−4^ M concentrations. One possible factor that might be attributed to the low inhibitive effect at 0.25 h exposure time in almost all sugars could be the ability of oysters to close their shells in response to stress [42], thereby, nullifying their inhibitive effect on the ability of the larvae to discriminate the presence of a conspecific cue. Although, it is interesting to note that even in this short duration, lactose has shown an inhibitive effect in the settlement at a relatively lower concentration, suggesting yet an unreported role this sugar might play in settlement induction of *C. gigas*. At 2 h exposure time, while only GlcNAc, lactose, and Neu5Ac were significantly inhibitive, all the other sugars, i.e., αMDM and Maltose, showed a lower settlement trend in larvae in the presence of a conspecific cue, CgSE. It was observed that regardless of sugar type, all larvae upon contact with a sugar solution, resumed swimming activity after more than an hour of exhibiting closed-shell behavior. It is possible that through this period, these dissolved sugars could have bound to the larval external chemoreceptors, hence, interfering with lectin-mediated processes involved in recognizing settlement inducing cues. The observation that larvae subjected to a 24 h co-exposure with sugar and conspecific cue were not significantly inhibited to settle in all sugar types could be attributable to the ability of oysters to produce high amounts of mucus under diverse forms of stresses [43]. Mucus contains mucoproteins associated with carbohydrates that are heavily glycosylated (up to 90% of carbohydrate) and present short carbohydrate chains whose charges are slightly negative [43]. Mucus matrices have been found to also contain repetitive highly sulfated polysaccharide, and have been shown to play a role in cleaning body surfaces with unwanted substances [43]. In this study, an elevated mucus production could have been initiated as the larvae were exposed to dissolved sugars for a prolonged duration and might have competitively bound carbohydrate moieties on the surface of the larval external chemoreceptors. Moreover, the excessive dissolved sugars may have been subsequently washed out from its system. Hence, eliminating the possible interference of these sugars from negatively influencing the recognition of the settlement inducing cue from adult shell extract in *C. gigas*.

The results from the WGA-sugar binding experiments showed that blocking the carbohydrate moieties of CgSE-treated GF/C papers by dissolved GlcNAc sugars decreased larval settlement in *C. gigas* larvae in a concentration-dependent manner and was found to be inhibiting at 10^−4^ M, confirming the findings of Vazquez et al. (2014) on conspecific shell HCl extract [9]. They also reported in that study that this shell conspecific HCl extract showed increasing positive binding to WGA-FITC conjugate with increasing intensity as the concentration of the extract increased. Hence, one possible reason for this pattern could be that the increased sugar concentration on the GF/C paper could have competed for the carbohydrate-binding sites on the CgSE, such that it suppressed its settlement inducing effect on the larvae. An alternative explanation could be that the shell CgSE may also contain a WGA lectin-like receptor that could have specificity for the GlcNAc sugars. Foulon et al. (2019) have hypothesized that perlucin-like proteins containing C-type lectin domains, present in the periostracum of *C. gigas*, mediate linkage between the adhesive, secreted by the larvae during cementation, and the periostracum [44]. Whether this lectin, or any other lectin found in the shell matrix, may contain a WGA-sugar binding carbohydrate recognition domain and is directly involved in larval settlement induction, remains to be investigated. Another possible factor is that the excessive amount of dissolved GlcNAc sugars may have bound to an oyster WGA lectin-like receptor, thereby, decreasing its ability to identify the signal from the conspecific cue in the extract. In a previous study by Vasquez et al. (2014), they postulated that the conspecific shell glycoprotein-induced settlement of *C. gigas* larvae are also mediated by oyster WGA lectin-like receptors [9]. Vasquez et al. (2014) have demonstrated that treating oyster shell HCl extract-treated GF/C papers with WGA effectively inhibited larval settlement in a concentration dependent manner. However, its effect on the presumed settlement inducer in shell HCl extract was reversible, as shown by a significant increase in the larval settlement after co-treatment with increasing concentrations of GlcNAc sugars [9]. The interaction of lectins with sugars is reversible since their interaction does not result in the formation of covalent bonds [28]. A similar trend of lectin binding interaction on the larval settlement was also observed in this study. Also, we report for the first time the involvement of Neu5Ac sugars in inhibiting the settlement inducing activity of a conspecific cue from CgSE which also showed a similar concentration dependent inhibiting pattern as the GlcNAc sugar. Both GlcNAc and Neu5Ac residues are known to bind with Wheat germ agglutin (WGA) lectin [45,46] which could interact with carbohydrates from different monosaccharide specificity groups at the same combining site [28]. GlcNAc residues found in oligosaccharides are usually situated in an internal position while Neu5Ac is commonly found at the terminal end [28]. Another possible reason for the ability of WGA to identify Neu5Ac is due to the structural similarity of this monosaccharide to *N*-acetylglucosamine (GlcNAc) [28]. In this study, the addition of GlcNAc and Neu5Ac sugars competitively attenuated the inhibiting effect of WGA on the presumed settlement inducer on CgSE-treated GF/C papers. This evidence points to the possible involvement not only of GlcNAc sugars, but also that of Neu5Ac residues, present in the presumed larval settlement inducer from a conspecific cue in shells of *C. gigas*.

This study also shows evidence of dissolved GlcNAc sugar binding to WGA lectin-like receptors distributed on different *C. gigas* larval tissues-tagged by WGA-FITC conjugate. Observations under the epifluorescent microscope showed more intense binding of WGA-FITC conjugate on the foot, mantle, and velum tissues in the larvae treated with 10^−10^ M GlcNAc solution while at a higher concentration, 10^−4^ M, resulted in a weak binding effect. In contrast, Khandeparker et al. (2011) have demonstrated that as the sugar concentration increased, glucose-treated barnacles exhibited increasing glucose-binding fluorescence with FITC-conjugated lectin, Concanavalin A, on the third antennular segment [31]. Earlier studies have suggested that the lentil lectin-binding sugar chains (glucose and mannose) identified in the settlement inducing protein complex (SIPC) pheromone, and which serves as a cue for its gregarious settlement behavior, is produced by unicellular glands and is secreted onto the antennular discs [30,31,34,47]. In the present study, one possible factor that could be attributed to the weakly bound stain on the oyster larval tissues at a higher concentration, 10^−4^ M, could be the presence of an increased amount of mucus secretion. Although, no mortality was recorded in the sugar exposure assay, an increased sugar concentration may have caused stress on the larvae, prompting it to produce more mucus, in order to remove excessive amounts of unwanted compounds in the body. The presence of mucus may have competitively bound to sites on the larval tissues recognized by the WGA-FITC conjugate. In a previous study of Espinosa et al. (2010), they have shown that pre-incubating microalgae with mucus from the pallial organs of *C. gigas* significantly reduced the binding effect of commercial FITC lectins to their surface ligands found in the microalgae [48]. Moreover, it is interesting to note that the non-sugar treated larvae tagged with WGA-FITC conjugate, in this study, showed positive binding on the foot and mantle tissues, suggesting the presence of endogenous GlcNAc, Neu5Ac, GalNAc residues [28] on the surfaces of these tissues. Also, no fluorescence was observed on the velum of the non-sugar treated larvae. A possible explanation for this could be that provided by Foulon et al. [49], where they demonstrated that a gland found on the *C. gigas* larval foot, similar to that reported on *Ostrea edulis* [50,51], showed acidic and neutral polysaccharide or glycoprotein content, and was also reported to play a role during the crawling phase. Additionally, the presence of endogenous WGA-binding sugar found on the larval foot and mantle, in the present study, further confirms one of the hypotheses of Vasquez et al. (2014). Their hypothesis explains that, during exposure of pediveliger larvae with WGA lectin treatment, this lectin may have to bound to shell matrix glycoproteins in the internal organs and mucopolysaccharides on the foot of the larvae [50,52,53,54,55], and thus, somehow interfered with *C. gigas* larval settlement behavior [9]. Shell matrix proteins, which are made by the mantle, interact with each other or with polysaccharides or chitin to produce the shell framework [56]. The settlement inducing protein from the conspecific shells may be found in the mantle and other multiple organs in *C. gigas* [54,55,57]. Although this warrants further investigation. Also, an earlier study demonstrated that the mantle specifically, its mantle margin, was actively involved during shell cementation in the settlement phase by adpressing the shell margin onto the substrate [58]. Moreover, the new findings in this present study suggest that a WGA lectin-like receptor and its endogenous ligand are both found in the larval chemoreceptors (foot, mantle, and velum). At the same time, the conspecific cue from the shell CgSE is also hypothesized to contain both, a settlement inducing endogenous ligand, and an endogenous lectin-like receptor that may complementarily work together to allow the larvae greater selectivity during site selection. The presence of bound endogenous lectin and its binding carbohydrate moieties in both the shell extract and the larvae could provide multiple binding sites and ensure higher affinity, selectivity, and complementarity [28], which could be advantageous for oyster larval growth and survival.

## 4. Materials and Methods

### 4.1. Spawning and Larval Culture of C. gigas

Adult Pacific oysters (*Crassostrea gigas*) were purchased from Konagai Fisheries Cooperative, Nagasaki, Japan. The spawning method and larval culture condition used in this experiment have been described in detail by Vasquez et al. [8]. Adult *C. gigas* broodstock were maintained in net cages, suspended from a raft of Nagasaki Prefecture Fisheries Station, Nagasaki, Japan (129°51′ E; 32°43’ N). They were brought to the laboratory for collection of gametes. Alternatively, the broodstocks were maintained in a 30-L aquarium inside the laboratory and were fed once daily with a combination of *Chaetoceros gracilis* and an artificial feed for bivalves (M1, Nosan Corp., Kanagawa, Japan). Adults were kept at 20 ± 1 °C in a 30-L aquarium during the spawning season to suppress the natural spawning of the broodstock. During the winter season, the adults were maintained at 25 ± 1 °C to allow gonad development and maturation. Every other day, 100% of the water in the aquarium was changed. We used at least four different broodstocks for spawning to obtain larvae for the different settlement assays.

Gametes were collected after stripping the adult oysters. Eggs and sperm were separately suspended in 2 L glass beakers containing GF/C (Whatman glass fiber filter; pore size: 1.2 mm) filtered seawater (FSW) adjusted to 27 °C. Eggs were washed several times with FSW through repeated decantation and were then fertilized with a small volume of sperm suspension. Thirty minutes after artificial fertilization, fertilized eggs were collected in a 20 mm net, washed four to five times with FSW, and re-suspended in 2 L glass beakers containing FSW. Fertilized eggs were kept at 27 ± 1 °C in an incubator for 24 h. Then, the swimming straight hinged larvae were collected in a 40 mm net, gently washed with FSW, stocked in 2 L glass beakers, at an initial density of 5 larvae mL^−1^, and cultured in a water bath at 27 ± 1 °C.

*C. gigas* larvae were mass-reared in 10-L tanks and were fed with *Chaetoceros calcitrans* (10,000–50,000 cells/mL) from day 1 to day 5, fed a combination of *C. calcitrans* (25,000 cells/mL) and *C. gracilis* (25,000 cells/mL) from day 6 to day 10, and were then fed *C. gracilis* (50,000 cells/mL) from day 11 onward during the culture period. Cultures were kept in a dark environment and the water was renewed daily throughout the culture period. The salinity of seawater used was 32 psu. Larvae usually reached the pediveliger stage 17–18 days after fertilization. Pediveligers used in assays were between 20 days old and 28 days old after fertilization and ranged from 300–360 mm in shell length.

### 4.2. Shell Preparation and Matrix Extraction

Shells from freshly shucked oysters (*C. gigas*) were thoroughly cleaned and washed with tap water to remove adhering epibionts and traces of muscle tissues, and then dried. Shell chips (SC) were prepared as described by Vasquez et al. [8]. Dried shells were crushed with a hammer to pass through a 1.0 mm mesh screen then through a 0.5 mm mesh screen. Shell fragments that remained on the 0.5 mm mesh screen were collected and used as shell chips (SC). 

Shell matrix extraction was performed following a modified method of Liu et al. [59]. Shell chips (150 g) were decalcified with 1 L of 0.8 M Ethylenediaminetetraacetic acid (EDTA, pH 8.0) for 60 h at 4 °C with continuous agitation. The supernatant was collected by centrifugation at 10,000 × g for 60 min at 4 °C. It was subsequently followed by filtration through a 0.22 µm membrane filter (MF-Millipore, Cork, Ireland), then suspended in 3.5 kDa molecular cutoff dialysis tubing (ø 28.6 mm; 1.25 nm pore size; Japan Medical Science, Osaka, Japan). To remove excess EDTA, the sample was extensively dialyzed against distilled water at 4 °C for 3 days, until the final pH of the extract was the same as the initial pH of the distilled water. The crude *C. gigas* shell EDTA extract (CgSE) was lyophilized and the resultant powder was dissolved in as small a volume as possible of distilled water and stored at -20 °C until further use. The protein content of the extract was quantified by a BCA assay kit (Pierce, Thermo Fisher Scientific, Rockford, IL, USA).

### 4.3. Larval Substrate ‘Choice’ Settlement Assay

The crude shell EDTA extract (CgSE) was subjected to a larval assay at various protein extract amounts and substrate types: 0, 50 µg, and 100 µg; GF/C, polystyrene, and PVDF, respectively. Shell chips (SC) at 50 mg was used as the reference substrate. Detailed descriptions of the preparation of different substrates prior to use in assays have been reported [9], except for polystyrene, where CgSE was directly applied on the multi-well plates and dried at 37 °C. All settlement assays were carried out using polystyrene multiwell plates (6-wells; ø 34 mm x 17 mm height; Violamo, As One Corporation, Osaka, Japan). Ten larvae were released into each well plate filled with 10 mL filtered seawater (FSW) and was repeated twice using two different batches of larvae with three replicates at each trial (*n* = 6). Settlement was evaluated by the number of individuals that metamorphosed to post-larvae within 24 h. Post-larvae were confirmed under the microscope as individuals that secreted cement substances or those with post-larval shell growth. All succeeding larval settlement assays were conducted in a dark environment at 27 ± 1 °C in an incubator.

### 4.4. Treatment of Larvae with Sugars and Settlement Assay

Larvae were immersed in millipore-filtered seawater (0.22 µm) containing graded concentrations (10^−10^, 10^−8^, 10^−6^, 10^−4^ M) of dissolved sugars for 2 h [3] relative to those recorded in previous studies [3,4,5,6]. Monosaccharides (D-arabinose, D-fructose, D-galactose, D-glucose, D-mannose, D-xylose, N-acetylglucosamine (GlcNAc), N-acetylneuraminic acid (Sialic acid or Neu5Ac), α-methyl-D-mannoside) and Disaccharides (lactose, maltose, sucrose) were used (Nacalai Tesque, Kyoto, Japan except for D-galactose (Wako Pure Chemical Co., Osaka, Japan)). After 2 h immersion into the sugar solutions, the sugar treated larvae were washed 3 times in 1 L FSW. Ten sugar treated larvae were introduced into 6-well plates coated with CgSE and to 10 mL FSW. The CgSE-coated surfaces were prepared by inoculating the 6-well plates with CgSE at a protein concentration of 50 µg mL^−1^. Untreated larvae were also released into the well plates containing CgSE-coated surfaces and FSW as the control. The 24 h larval settlement assays were repeated twice using different batches of larvae with three replicates at each trial (*n* = 6).

### 4.5. Assay Protocol on the Effect of Exposure Time on Sugar and Conspecific Cues Induction of Settlement

The effect of exposure times on selected inhibitory sugars (GlcNAc, Neu5Ac, αMDM, lactose, maltose) at 10^−6^ and 10^−4^ M was examined. Exposure times were 0.25, 2, and 24 h. 

After a specific exposure time, the sugar-treated larvae were washed three times in 1 L FSW and were used in settlement assays, as described earlier.

### 4.6. Assay Protocol on WGA and Sugar (GlcNAc and Neu5Ac) Interaction

Wheat germ agglutinin lectin (WGA; Wako Pure Chemical Co., Osaka, Japan) is known to bind with N-acetylglucosamine (GlcNAc) and N-acetylneuraminic acid or Sialic acid (Neu5Ac) sugars. Lectin-sugar interaction between WGA-GlcNAc and WGA-Neu5Ac was investigated following the method of Vasquez et al. [9]. In brief, CgSE was inoculated on a GF/C filter paper (ø 47 mm, GF/C) and dried at 37 °C. Dried CgSE papers were immersed in solutions containing 50 µg mL^−1^ WGA lectin or in mixtures of 50 µg mL^−1^ WGA and different concentrations (10^−8^, 10^−6^, 10^−4^ M) of GlcNAc or Neu5Ac sugars for 2 h. After 2 h, treated CgSE papers were washed three times in 1 L FSW and then used in assays. CgSE papers that were not treated with the mixture of WGA and GlcNAc or Neu5Ac were also washed in the same manner and were used as the control. All GF/C filter papers were fastened to the bottom of the well using double-sided adhesive tape. In a 6-well plate containing different coated and non-coated GF/C filter papers, ten larvae were released into each well filled with 10 mL FSW. A 24 h larval settlement assay was carried out using three replicates for each treatment (*n* = 3).

### 4.7. Treatment of Oyster Larvae with Fluorescein Isothiocyanate-Conjugated WGA

To examine the distribution of GlcNAc sugar bound on *C. gigas* larval tissues, the larvae were stained with FITC-conjugated WGA (Sigma-Aldrich, Rehovot, Israel). The larvae were immersed in FSW containing 10^−10^ and 10^−4^ M GlcNAc solutions for 2 h and subsequently rinsed three times in 1 L FSW, dyed for 5 min in a solution of FITC-WGA diluted in DW at 0.5 mg mL^−1^. After incubation for 5 min, the larvae were rinsed by filtered seawater; then immersed in 7.5% MgCl_2_ solution in a polystyrene 6-well plate and observed using an epifluorescence microscope (Excitation wavelength (437–460 nm (448 nm)); Emission wavelength (472 nm); Nikon Eclipse Ts2, Nikon Corporation, Tokyo, Japan). The use of MgCl_2_ has been reported as an effective anesthetic that renders an animal in a complete loss of muscle tone, thus, subjecting it to a relaxed state suitable for physiological observations [60]. The untreated larvae exposed to WGA-FITC were used as control. To check for autofluorescence, untreated larvae that were not dyed with WGA-FITC were also compared.

### 4.8. Statistical Analysis

The settlement percentage (%) was calculated based on the number of settled larvae over the total number of larvae in each well multiplied by 100. These percentages were presented as arithmetic means with standard error means (SEM). Data were analyzed using quasi-binomial generalized linear models (GLM). Wald test [61] was used for pairwise comparisons. All statistical tests were performed in RStudio (R-project.org, version 4.0.3).

## 5. Conclusions

This study reports a conspecific cue from the *C. gigas* shell-EDTA extract, which exhibits a concentration-dependent settlement, thereby, inducing activities on all tested substrates, i.e., GF/C, polystyrene, and polyvinylidene difluoride. It also demonstrates the involvement of carbohydrate-lectin interaction and the important role played by sugar compounds in settlement site selection by *C. gigas* larvae. Sugar compounds can influence the ability of the larvae to identify and discriminate suitable substrata in response to chemical cues in either inhibiting or enhancing lectin-mediated processes involved in biorecognition. Sugars in the absence of a conspecific cue, *C. gigas* adult shell extract, did not promote settlement whereas, in the presence of the cue showed varied effects, most of which were found inhibitory at different concentrations. The binding affinity of selected inhibitory sugars such as GlcNAc, Neu5Ac, and lactose is influenced not only by its concentration but also by exposure time, with 2 h duration eliciting the maximum inhibitive effect on oyster larval settlement in the presence of a conspecific cue. Blocking of WGA-binding sites on the shell extract significantly reduced its settlement induction efficiency and the presence of GlcNAc sugar binding to WGA lectin-like receptors distributed on different *C. gigas* larval tissues-tagged by WGA-FITC conjugate was confirmed. New findings in this present study suggest that a WGA lectin-like receptor and its endogenous ligand are both found in the larval chemoreceptors (foot, mantle, and velum). At the same time, the conspecific cue from the shell CgSE has also been hypothesized to contain both a settlement-inducing endogenous ligand, as well as an endogenous lectin-like receptor that may complementarily work together to allow the larvae greater selectivity during site selection. This present work provides new insights into how carbohydrate-lectin interaction could play an important role in the natural environment when larvae encounter different glycoproteins adsorbed to surfaces and are also exposed to dissolved cues. It opens new possibilities for further analysis to help us understand the underlying mechanisms that shape population structures and chemical signaling.

## Data Availability

All the data used in this study have been provided in the main text.
